# Expression and therapeutic significance of estrogen receptor β in malignant pleural mesothelioma

**DOI:** 10.4155/fsoa-2017-0007

**Published:** 2017-02-17

**Authors:** Giulia Pinton, Laura Moro

**Affiliations:** 1 Department of Pharmaceutical Sciences, University of Piemonte Orientale, Novara, Italy

**Keywords:** estrogen receptor β, malignant pleural mesothelioma, prognosis, therapy

Malignant pleural mesothelioma (MPM) is an asbestos-associated aggressive cancer that arises from the mesothelial lining of the pleura. Although the use of asbestos has been banned in many countries, the potentially hazardous exposure to asbestos is still present with locally high incidences of mesothelioma [1]. Chemotherapy for MPM has remained unchanged since 2003 [2], when the combination of cisplatin and pemetrexed became the standard first-line therapy for patients who are not suitable for aggressive surgery, or in whom chemotherapy is recommended as part of a multimodality regimen. Unfortunately, the disease recurs in over 80% of the patients at the 2-year follow-up mark. In the second-line setting, a variety of cytotoxic agents have been tested, either as single or combined treatment [3], but none of these have been adopted as standard therapy. Advances in the understanding of MPM biology bring, however, hope that novel agents will be identified that improve patient outcomes.

The observation that women diagnosed with MPM respond better to treatments and live longer than men, prompted us to assess the role of estrogens and their receptors in this cancer.

Estrogens play important roles in several physiological and pathological processes. The existence of an estrogen receptor (ER) was demonstrated in 1958 [4] and the corresponding gene was cloned in 1985. It was considered the only receptor responsible for mediating estrogen action until the gene coding for a second ER subtype, ERβ, was cloned in 1996 [5]. This second receptor is expressed in many human tissues, including some that traditionally are considered non target for estrogens. One of the more interesting observations about ERβ is that it exerts antiproliferative effects in different preclinical *in vitro* and *in vivo* models of human cancers [6–8].

We analyzed the expression of the two ER subtypes, α and β, in tumor biopsies from MPM patients and in normal pleura from healthy controls [9]. Nuclear ERβ immunoreactivity was detected in normal pleura and in the majority of the 78 analyzed MPM samples, although with reduced presence and intensity, compared with normal pleura. The cumulative probability of survival after 2 years of follow-up was 80% for subjects with high ERβ expression versus 31% for subjects with negative of low ERβ expression (p = 0.02, log-rank test).

Importantly, multivariate analysis of overall survival demonstrated the prognostic significance of ERβ staining. Different from other lung cancers, none of 78 MPM biopsies or normal pleura stained positive for ERα. MPM cells represent, therefore, a powerful model to study the ERβ role and function.

## Key findings

### ERβ inhibits MPM cell proliferation

We hypothesized that the observed expression of ERβ in MPM samples and longer survival of MPM patients could reflect its tumor-suppressor properties. To test this hypothesis, we performed studies *in vitro* providing evidence that ERβ plays a role in the control of MPM cell proliferation by downregulation of cyclin B1 and survivin, causing a G2-M-phase arrest in cell cycle progression [8]. Then, we performed a meta-analysis of global gene expression profiles of 93 MPM samples to identify an *ESR2* (ERβ-coding gene) signature.

Among genes downregulated in tumors expressing high levels of *ESR2* we identified *SDHB*, which encodes the B subunit of the succinate dehydrogenase (complex II of the mitochondrial respiratory chain). We demonstrated that ERβ, activated by overexpression or by selective agonist stimulation, impaired complex II and IV activity compromising mitochondrial oxidative phosphorylation. This led to reduced mitochondrial ATP production, increased glycolysis dependence and decreased cell proliferation.

Furthermore, the reported *in vitro* effects on the mitochondrial respiratory chain complexes translated to an *in vivo* mesothelioma tumor model treated with the ERβ-selective agonist KB9520 [10].

### ERβ affects epithelial–mesenchymal transition in MPM

MPM may also be a powerful model for studying epithelial–mesenchymal transition (EMT) *in vivo* [11]. Aside from the location and stage of the disease, mesothelioma is also categorized by cell type. According to the WHO classification, MPM is subclassified as epithelioid (mostly composed of epithelial-shaped cells), sarcomatoid (mostly composed of spindle-shaped cells) or biphasic (composed of both cell types). The morphological patterns of MPM are therefore likely to be the outcome of different phases in the EMT process. Each cell type responds differently to treatment and has an important effect on a patient's prognosis. Mesothelioma tumors made up of epithelioid cells are the most treatable and patients with this cell type have the best prognosis.

We demonstrated that re-expression of ERβ in ER-negative cells originating from biphasic MPM conferred a more epithelioid phenotype, decreased capacity for anchorage-independent growth and down-modulated proliferative signal transduction pathways. Conversely, ERβ knockdown in ER-positive cells conferred a more invasive phenotype, increased anchorage independent growth and elevated EGFR-coupled signal transduction pathways [12].

The possibility to reverse the more aggressive biphasic mesothelioma histotype by targeting ERβ with a selective agonist could, therefore, represent a novel and important treatment strategy to stabilize this aggressive disease, with manageable toxicity.

### ERβ is expressed by ER-negative MPM cells in hypoxia

Hypoxia is a common feature in MPM. A pilot study performed with [F-18] fluoromisonidazole PET-CT analysis has provided evidence of significant areas of hypoxia in MPM-dominant tumor masses [13]. Another study has described carbonic anhydrase IX positivity, proposed to serve as a surrogate marker of hypoxia, to be predominant in epithelioid MPMs, while not expressed in sarcomatoid and sarcomatoid areas of biphasic MPMs [14]. Differently from other tumors in which the hypoxic condition induces EMT and invasion, we have described that hypoxia causes, in cells derived from biphasic MPM, the switch from spindle to epithelioid phenotype with increased E-cadherin expression and reduced growth rate [15]. Changes in epigenetic marks, including lysine methylation of histones, have been observed in development and in disease states where hypoxia is known to be an important feature. Histone-lysine methylation is dynamically regulated by histone methyltransferases and histone demethylases (KDMs). We reported a strong positive correlation between the expression of the KDM6B and ERβ in MPM tumors and cell lines. We described that, in hypoxia, the HIF2α–KDM6B axis induced an epithelioid morphology and ERβ expression in biphasic MPM cells with ER-negative phenotype. ERβ was also transiently expressed by ER-negative cells cultured as spheroids *in vitro* or as tumor mass *in vivo* when hypoxic conditions occurred. Importantly, ERβ expression and tumor-suppressive function were maintained by selective ligand activation [16].

### ERβ activation increases sensitivity of MPM to the standard of care

We investigated the possibility of an additive or synergistic effect between the ERβ selective agonist KB9520 and the standard of care (cisplatin/pemetrexed) for treatment of MPM. We showed that KB9520 acted as a chemosensitizer through activation of ERβ, increasing the antitumorigenic efficacy of cisplatin or the cisplatin/pemetrexed combination. Treatment with KB9520 in combination with cisplatin/pemetrexed *in vivo* had greater efficacy than either treatment alone and caused a significantly reduced tumor burden compared with vehicle-treated animals [17]. Importantly, KB9520 had no cytotoxic effect per se and reduced cisplatin toxicity in ERβ-expressing non malignant mesothelial cells. Thus, KB9520 may increase the sensitivity of MPM tumors to the standard of care in patients and perhaps result in higher response rates, without adding toxicity.

## Implications & future perspective

Drugs that selectively target ERβ, being free from the undesired ERα-promoted proliferative effects on breast and uterus, might be safer than non selective estrogens [18]. Several synthetic and natural ERβ-selective agonists have been identified that have exhibited promising antitumorigenic activity in preclinical cancer models. Another important observation is that ERβ selective agonists can increase the expression of ERβ in cells where its expression has been downregulated. Therefore, drugs with selectivity for ERβ might prove promising in the development of novel, targeted therapies for the clinical management of human cancers.
